# Functional Outcome Estimation of Calcaneum Fractures Treated by Open Reduction and Internal Fixation with Plate and Screws in A Tertiary Centre: A Descriptive Cross-sectional Study

**DOI:** 10.31729/jnma.5273

**Published:** 2020-09-30

**Authors:** Rajeev Dwivedi, Mandir Khatri, Arjun K C

**Affiliations:** 1 Department of Orthopedics, Lumbini Medical College and Teaching Hospital, Tansen, Palpa, Nepal

**Keywords:** *calcaneus*, *intra-articular fractures*, *treatment*

## Abstract

**Introduction:**

Calcaneum fractures constitute about 60% of all tarsal bone fractures. Intra-articular fractures account for 70% of all calcaneal fractures. There are controversies regarding the operative treatment of calcaneum fractures. Therefore this study aimed to estimate the functional outcomes of calcaneum fractures treated by open reduction and internal fixation with plate and screws.

**Methods:**

This descriptive, cross-sectional study was carried out at the tertiary care center in the western region of Nepal among the patients with displaced intra-articular calcaneum fractures from February 2017 to July 2020 after approval from the Institutional review committee. Convenient sampling was done to reach the sample size. Fifteen cases were included in the study. Data were recorded in proforma and Data analysis was done in the statistical package for social sciences. The American Orthopedic Foot and Ankle Society Hindfoot score was used to assess the final outcome.

**Results:**

According to the American Orthopedic Foot and Ankle Society hindfoot scores, there were five excellent (33.33%), seven good (46.66%), two fair (13.33%) and one poor (6.66 %) results.

**Conclusions:**

In displaced intra-articular calcaneum fractures, open reduction and internal fixation with plates and screws result in a good number of satisfactory outcomes with very few unsatisfactory results. Hence it can be a better option of treatment in displaced intra-articular calcaneum fractures.

## INTRODUCTION

Although calcaneal fractures are uncommon, comprising approximately 2% of all fractures.^1–3^ They constitute about 60% of all tarsal bone fractures.^1^ Mostly they are due to high-energy axial trauma, mainly due to falls from a height.^1,2,4,5^ Intra-articular fractures account for 70% of all calcaneal fractures. They are the most challenging and outcomes are unpredictable.^1^ There is no consensus between surgical and conservative treatment in terms of outcomes.^2^

Due to difficult terrain and frequent motor vehicle accidents in this area, the calcaneal fracture is the most common tarsal bone fracture managed at our center. We are managing these injuries by both operative and non-operative methods. Data regarding outcomes of operative treatment of calcaneum fracture is sparse. Therefore, we decided to conduct this study.

This study aimed to estimate the functional outcomes of calcaneum fractures treated by open reduction and internal fixation with plates and screws.

## METHODS

This descriptive cross-sectional study was conducted at the Department of Orthopedics, Lumbini Medical College, and Teaching Hospital from February 2017 to July 2020 for over three and a half years. This study was approved by the Institutional Review Committee (IRC-LMC 18-D/020) of LMCTH, Tansen, and Palpa, Nepal. Both the verbal and written informed consents were taken from each of the participants.

The study population is patients who have been admitted to this institution with a diagnosis of calcaneum fracture. Hospital records including case files, operation details, and discharge records were reviewed to enroll the patients for study after due permission from concern authority. Patients aged 18 years and above with displaced intra-articular calcaneum fracture managed with open reduction and internal fixation by calcaneum locking plates and screws were included in the study. Undisplaced calcaneum fractures, compound fracture, patients with impaired sensation in lower limbs, were excluded from the study. The initial evaluation before operation comprised plain radiographs, lateral and axial views of calcaneum, as well as computed tomography (CT) scans. Calcaneal fractures were classified according to the Sanders classification based on CT scans.^6^

Age, sex, mechanism of injury, laterality, type of fracture as per Sander's classification, type of surgery, postoperative wound complication, time elapsed between the injury to surgery were recorded. Patients who visited for follow up themselves and patients who return for follow-up after telephonic communication were examined and final outcomes were measured with validated the American Orthopedic Foot and Ankle Society (AOFAS) Hindfoot questionnaires (Scores).^7^ The AOFAS hindfoot score system assesses the intensity of pain and functional disability and mainly includes nine aspects: pain 40 points, function 50 points which include, maximum walking distance (blocks), walking surfaces, gait abnormality, sagittal motion (flexion plus extension), hindfoot motion (inversion plus eversion), ankle-hindfoot stability (anteroposterior, varus-valgus), and alignment 10 points. The score had a maximum of 100 points (best possible outcome). The results are considered as excellent when the scores ranged from 90 to 100, good when between 80 and 89, fair when ranging from 70 to 79 and bad when below 70. At final follow-up, Lateral and axial x-rays of calcaneum were taken for radiological evaluation, Bohler's angle was measured in plain lateral radiograph of calcaneum, 25-40 degrees is considered as normal value, an angle is reduced in displaced intra-articluar fracture.^8^ Radiographs showing complete bridging callus formation and the absence of radiolucent lines were used to define bone healing.

A total of 15 patients (15 heels) with displaced intraarticular calcaneum fractures meeting the inclusion criteria came for final follow up. Two patients had bilateral calcaneum fractures. Since, contralateral fracture was undisplaced it was excluded from the study. Undsiplaced fractures were managed by the non-operative method. All patients with displaced intra-articluar fractures were treated by open reduction and internal fixation. In all patients' lateral extensile approach was used for exposure of fracture and in all cases low profile calcaneal locking plate and locking head screws were used.

Convenience sampling was done and the minimum sample size was calculated using the formula,

$$\begin{array}{ll} \text{n} & \text{= }{\text{Z}}^{\text{2}}\times \text{p}\times \text{q}/{\text{e}}^{\text{2}}\\ & \text{= }{\left(1.96\right)}^{\text{2}}\times 0.02\times (1-0.02)/{\left(0.1\right)}^{\text{2}}\\ & \text{= }7.52 \end{array}$$

Where,

Z = 1.96 at 95% CI.
p = prevalence of calcaneum fracture 2 % (references 1-3),
q = 1-p
e = margin of error, 10%

The minimum sample size was calculated to be 8. Selection bias and interpretation bias was minimized as possible. Data were recorded in the proforma form. The data was then coded and entry was done in the statistical package for the social sciences (SPSS) version 16.0. The data was processed and analyzed by using simple descriptive statistics; in terms of percentage and frequency.

## RESULTS

The characteristics of study participants are shown (Table 1).

**Table 1:** Showing Characteristics of study participants (n=15)

Characteristics (n = 15)		Findings
Age in years {standard deviation(SD)} range 18-50 years		32.4 (10.44)
Male: female (Laterality (Right:Left) 9:6”		13:2
Mechanism of injury Fall from height		15
Fractures as per Sander’s classification	Type II	8 (53.33%)
	Type III	6 (40%)
	Type IV	1 (6.66%)
The average duration between the injury to surgery(SD)		10.87 (1.68) days
		Range 8 to 15 days
Mean AOFAS hindfoot score(SD)		82.33 (11.01)
Mean Bohler’s angle (SD)		26.40 (5.98) degree
Mean Follow-up duration in months (SD)		14.53 (8.63)

According to the AOFAS hindfoot scores, there were five excellent (33.33%), seven good (46.66%), two fair (13.33%) and one poor (6.66 %) results. The outcome of patients in a different type of Sander's fractures is shown (Table 2).

**Table 2:** Outcomes of patients in a different type of Sander’s fractures.

Sander’s classification	Functional outcome n (%)			
	Excellent	Good	Fair	Poor
Type II	4 (50)	3 (37.5)	1 (12.5)	0
Type III	1 (16.66%)	4 (66.66%)	1 (16.66)	0
Type IV	0	0	0	1 (100%)

At the final follow-up out of 15 patients, 4 (26.66%) had Bohler's angle <20, and 11 (73.33%) patients had Bohler's angle of 21-40. Comparison of outcomes in Bohler's angle less 20 and between 20 to 40 are shown (Table 3). All fractures were united at final evaluation. Out of 15 patients, 4 patients had complications in the form of delayed wound healing which improved with regular dress and antibiotics.

**Table 3:** Comparison of outcomes in Bohler’s angle less than 20 and between 20 to 40.

Postoperative Bohler’s angle	The outcome as per AOFAS hindfoot score	
	Excellent and good n (%)	Fair and poor n (%)
<20	2 (16.66)	2 (66.66)
20-40	10 (83.33)	1 (33.33)

## DISCUSSION

Though there is a conflict regarding operative versus nonoperative treatment in displaced intra-articular calcaneum fractures, there are studies supporting operative treatment over nonoperative treatment. With the advancement in implants and wound care, there is a trend towards the surgical treatment of displaced calcaneum fractures. In the present study, overall outcomes of open reduction and internal fixation in displaced intra-articular calcaneum fractures was satisfactory, average AOFAS score at final follow-up was 82.33 (SD, 11.01), with 12 out of 15 cases (80%) having excellent to good results and 2 (13.33%) and 1 (6.66%) had fair and poor results respectively. There are various other studies, either they have used similar (AOFAS score) or different scoring systems, they have found more favorable results in the operative treatment of calcaneum fractures similar to our study.

Almeida, et al,^9^ studied 44 patients with intra-articular calcaneal fractures, managed by open reduction and internal fixation with Reconstruction or Y plate, and outcomes were assessed with AOFAS score. At the final follow up, excellent results were achieved in 31.8%, good results in 11.4%, fair result in 29.5%, and poor result in 27.3%. They concluded open reduction and internal fixation can be recommended as a very good alternative to conservative treatment in intra articular calcaneal fractures.

Santosha, et al,^10^ evaluate the functional outcome after open reduction and internal fixation of displaced intra-articular fractures of the calcaneum by locking calcaneal plates in 24 patients. According to the AOFAS score results were excellent in 43.3% of the patients, good in 33.3%, fair in 10%, and poor in 13.3% of patients. They concluded Open reduction and internal fixation of intraarticular fractures of the calcaneum with locking calcaneal plate gives good results.

In a study conducted by Palange, et al.^11^ 20 patients out of 30 had good results, 7 patients had fair results while the remaining 3 patients had poor results. Postoperatively, wound complications were seen in 2 patients who settled after debridement and medications. No other complication was encountered. In the study by Rak et al.^12^ the overall results according to the AOFAS score were good or excellent in 30/34 (85%) in patients treated by open reduction and fixation with calcaneum locking plate and screws.

Shresth R, et al.^13^ evaluated the outcome of calcaneum fracture using Maryland Foot Score (MFS) managed by open reduction and internal fixation with Locking Branched Calcaneal Plates through the extensile lateral approach. Seventeen cases (77.13%) had good, four cases (18.2%) had fair, and one case (5.5%) had a poor outcome score, similar to our study. They concluded displaced intra-articular calcaneal fractures treated operatively with open reduction and internal fixation with locking branched calcaneal plates through the extended lateral approach, with proper planning of operation and surgical techniques in soft-tissue handling, results in good clinical as well as radiological outcomes.

In the present study, more number of excellent and good cases had Bohler's angle between 20 to 40 degrees. Kulkarni, et.al.^14^ compared operative and conservative treatment in displaced intraarticular fractures of calcaneum (n = 15 in each group). They evaluated restoration of Bohler's angle along with Creighton-Nebraska (C-N) score for functional outcome. At the end of 12 months, a relatively better functional outcome was observed in displaced and comminuted fractures in plating, provided that the Bohler's angle was restored. They emphasized Post-treatment Bohler's angle has prognostic importance in functional outcome.

Makki, et al.^15^ carried out a retrospective review of 47 intra-articular fractures of the calcaneum treated by open reduction and internal fixation. According to AOFAS score there were 18 excellent (38.3%), 17 good (36.2%), three fair (6.3%) and nine poor (19.2%) results. In this study restoration of Bohler's angle was associated with a better outcome. They opine osteosynthesis should be considered for intra-articular fractures of the calcaneum to restore the shape of the hindfoot and Bohler's angle.

Against the result of our study, there are studies where results are not favorable for open reduction and internal fixation. Buckely, et.al.^16^ studied 471 displaced intraarticular calcaneal fractures were they used the Short Form-36 (SF-36, a general health survey) and a visual analog scale (a disease-specific scale) for outcome evaluation. The outcomes after nonoperative treatment were not found to be different from those after operative treatment; the score on the SF-36 was 64.7 and 68.7, respectively (p= 0.13), and the score on the visual analog scale was 64.3 and 68.6, respectively (p = 0.12).

Jarvholm, et al.^17^, compared close reduction with open reduction, they concluded Open reduction of the intraarticular fracture of the calcaneus may provide stability, allowing early motion and eventually improved subtalar function. However, postoperative complications are common, the overall results of open and closed treatment are almost equal, and the Primary operation of the fractured calcaneus should therefore rarely be indicated.

In a randomized controlled trial conducted by Griffin, et al.^18^ operative treatment compared with non-operative treatment showed no symptomatic or functional advantage after two years in patients with typical displaced intra-articular fractures of the calcaneus, and the risk of complications was higher after surgery. They concluded operative treatment by open reduction and internal fixation is not recommended for displaced intraarticluar calcaneum fractures.

A meta-analysis conducted by Wei, et al. ^19^ to compare operative versus nonoperative treatment of displaced intra-articular calcaneal fractures has shown operative treatment of displaced intra-articular calcaneum fracture may lead to a higher incidence of complications but has better anatomical recovery when compared with nonoperative treatment.

The limitations in our study were the small number of cases, the shorter time of follow-up, and no comparison with cases that are treated conservatively.

## CONCLUSIONS

In displaced intra-articular calcaneum fractures, open reduction and internal fixation by extended lateral approach with calcaneum locking plates and screws result in a good number of satisfactory outcomes with very few unsatisfactory results. Hence, it can be a better option of treatment in displaced intra-articular calcaneum fractures.

## Conflict of Interest

**None.**
